# Pelvic Organ Prolapse: Current Challenges and Future Perspectives

**DOI:** 10.3390/jcm14207313

**Published:** 2025-10-16

**Authors:** Anna Padoa, Andrea Braga, Sharon Brecher, Tal Fligelman, Giada Mesiano, Maurizio Serati

**Affiliations:** 1 Department of Obstetrics and Gynecology, Chaim Sheba Medical Center, Ramat Gan 52621, Israel; sharon.brecher@gmail.com (S.B.); talf62@gmail.com (T.F.); 2 Faculty of Medical & Health Sciences, Tel Aviv University, Tel Aviv 6997801, Israel; 3 Department of Obstetrics and Gynecology, EOC, Beata Vergine Hospital, 6850 Mendrisio, Switzerland; andrea.braga@eoc.ch; 4 Faculty of Biomedical Sciences, Università della Svizzera Italiana, 6900 Lugano, Switzerland; 5 Department of Obstetrics and Gynecology, Del Ponte Hospital, University of Insubria, 21100 Varese, Italy; giadamesiano01@gmail.com

**Keywords:** pelvic organ prolapse, patient-centered treatment, native tissue repair, sacrocolpopexy, vaginal pessary, quality of life, patient-reported outcomes

## Abstract

Pelvic organ prolapse (POP) affects millions of women around the world, with age-standardized prevalence rates of 2769 per 100,000 women in 2021. Although it greatly affects quality of life (QoL), only 18–50% of women experiencing this issue seek medical attention, largely due to a lack of knowledge, misunderstandings about the condition, and obstacles to accessing healthcare. This narrative review explores the progression of POP management towards a focus on patient-centered care, highlighting the importance of personalized treatment strategies that prioritize patient-reported outcomes (PROs) over solely anatomical factors. The approach to treatment has transitioned from being centered on anatomy to focusing on the patient, emphasizing the relief of symptoms and enhancement in QoL. Existing research indicates that monitoring without intervention is advisable for asymptomatic patients, as long-term studies have revealed that up to 40% of women experience stable or improved prolapse over a period up to 60 months. Pessary treatment has a fitting success rate above 90% and a treatment persistence rate of 60%, providing an effective non-surgical option for management. The approach to selecting surgical treatments has progressed to prioritize sufficient apical support as a key factor for achieving lasting results. For primary POP, native tissue repair (NTR) is now recommended as the first-line surgical option. Mesh-augmented repairs are used only in certain high-risk situations, whereas sacrocolpopexy offers the best anatomical stability for particular cases, such as those involving post-hysterectomy prolapse and recurrences. Contemporary POP management involves personalized, patient-focused decision-making that emphasizes addressing symptom severity and functional objectives rather than solely aiming for anatomical precision. The evidence suggests that NTR should be the primary surgical approach, while other procedures should be reserved for specially chosen patients. Success should primarily be evaluated based on PROs instead of anatomical factors, ensuring that treatments align with each patient’s preferences and expectations while reducing complications.

## 1. Introduction

Pelvic organ prolapse (POP) is the anatomical descent of pelvic organs resulting in protrusion into or beyond the vaginal canal. Professional societies recognize that the diagnosis should be based on either patient-reported symptoms or objective findings on examination [1,2,3]. The global age-standardized prevalence rate of POP in 2021 was 2769 per 100,000 women, with the highest prevalence in women aged 80 and older: the absolute number of cases has increased due to population aging, with projections estimating that by 2036, there will be 156 million women globally affected [4,5,6]. Prevalence estimates vary between 1 and 31% in symptom-based surveys, to 10–50% in physical examination-based studies, and up to 65% in studies with combined approaches [7]. Risk factors for POP include increasing age, higher parity, vaginal delivery, assisted delivery, higher birthweight, elevated body mass index, and levator ani muscle defects [8,9]. Considering recurrent cases of POP after surgery, higher preoperative POP stage and younger age are significant risk factors [5,6,7,8]. In summary, the absolute burden is rising due to demographic shifts, and the condition remains a major public health issue, particularly in aging and underserved populations [1,2,3].

This narrative review aims to provide guiding principles to assist general practitioners, gynecologists, and pelvic floor medicine experts in counseling women with POP. The review outlines evidence-based indications and potential contraindications for each treatment strategy, while acknowledging regional and individual surgeon practice variability.

## 2. POP Treatment Paradigm: The Patient-Centered Approach

Women with POP may choose to be evaluated by a healthcare provider to confirm the diagnosis, assess the severity, identify potential association with pelvic floor symptoms (such as urinary, bowel, or sexual dysfunction), and discuss individualized management options. However, only 18–50% of women with pelvic floor dysfunction (PFD) seek medical care [10], often due to insufficient knowledge and misperceptions, such as belief that such conditions are a normal part of aging, lack of awareness of available treatment options, or fear of the need for invasive procedures [11]. A cross-sectional study among 431 community-dwelling women found considerable deficits in knowledge about PFD in women of all ages, races, and socio-economic levels: only 48% of the study cohort demonstrated proficiency in POP knowledge [12]. It is not uncommon for women to present in emergency settings due to anxiety about POP symptoms such as abnormal vaginal bleeding or the sudden awareness of a vaginal bulge, often accompanied by fear of malignancy. A recent retrospective study [13] reported characteristics of 56 women presenting at the emergency department due to POP: Black women and women with public insurance only (i.e., limited access to routine gynecological care) were overrepresented in this cohort. The main symptoms were “vaginal bulge” and “pelvic pain”, and only 42% of the study cohort returned for follow-up at a urogynecology clinic as recommended at discharge. The above studies underscore that improved framing, awareness, and self-efficacy must be promoted among women at risk for POP, despite the scant evidence on patient perceptions of this condition. The combination of limited knowledge and resources, embarrassment to disclose intimate complaints, and the complex power dynamics often at play between women and providers may result in profound vulnerability of women affected, exposing them to a disturbing combination potential underdiagnosis, dismissal, or overtreatment. Adoption of a patient-centered approach in POP treatment emphasizes shared decision-making, individualized assessment of symptoms and goals, and the use of validated patient-reported outcome (PRO) measures to guide therapy and monitor response. Given that it is not frequent that POP impacts general health, but in a majority of women it may significantly compromise quality of life (QoL), all management decisions should be driven by the patient’s bother, preferences, and goals, rather than solely by anatomic findings. For asymptomatic women, education and reassurance are appropriate, and a “watchful waiting” approach can be adopted, scheduling periodic appointments to allow monitoring of symptom development and enable timely treatment [14]. Symptomatic women should be offered both non-surgical and surgical options, with counseling on risks, benefits, and expectations, thus aligning treatment with patient preference and their tolerance for risk, invasiveness, and potential complications [15].

## 3. Treatment Approaches and Considerations

**Figure 1:**
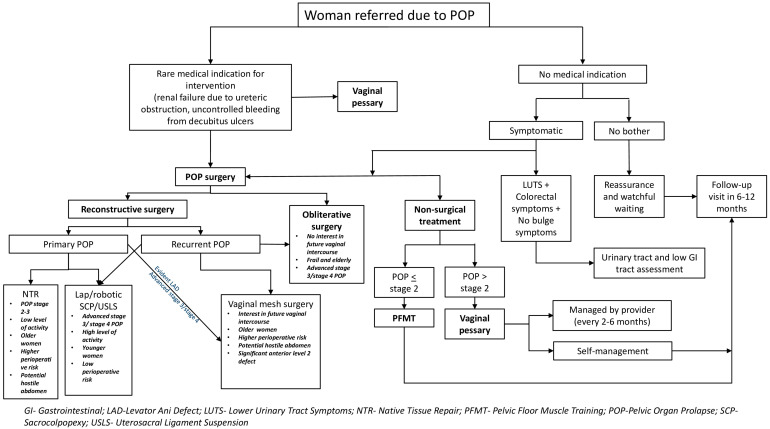
Suggested algorithm for management of pelvic organ prolapse.

A suggested algorithm for management of POP based on women’s needs, goals, and preferences is outlined in Figure 1.

### 3.1. Watchful Waiting—For Whom and How?

Observation without intervention should be the recommended approach for women with asymptomatic or minimally symptomatic POP, as most women will not experience rapid progression or complications. Usually, the natural history of POP is characterized by slow progression, with many women experiencing little or no change over time. Prospective cohort studies indicate that, over several years, the majority of women with symptomatic or asymptomatic POP will have stable or even improved prolapse, while a minority will experience progression. Three longitudinal cohort studies [16,17,18] with follow-up ranging between 16 and 60 months, showed regression of POP stage in up to 40% of women, while only 6–20% had progression of the POP-Q stage, with or without worsening of symptoms. Based on these data, the American Urogynecologic Society (AUGS) and the American College of Obstetricians and Gynecologists (ACOG) both state that watchful waiting is an evidence-based and safe option for many women with non-bothersome POP [2,19].

### 3.2. Vaginal Pessary Treatment: For Whom, How, and When

Vaginal pessaries are mechanical devices inserted into the vagina to reduce the prolapsed uterus or vaginal walls, and constitute the only highly effective long-term non-surgical treatment for POP. Typical candidates for pessary treatment are women who have not completed childbearing and frail patients at poor operative risk. The option of pessary management should be brought to knowledge of all women with symptomatic POP, provided there are no medical indications for surgical treatment besides the prolapse. Recent systematic reviews have demonstrated high efficacy of pessary treatment for improving POP symptoms and QoL [20,21,22]. However, long-term adherence due to factors like improper fit, discomfort, and lack of self-management support may decrease compliance. While a significant obstacle to successful pessary treatment is incorrect fitting, a recent RCT [23] has indicated successful pessary fitting can be achieved in 92% of women, with treatment persistence of 60%. In order to guarantee correct fitting, it is best for urogynecology clinics to purchase one of the commercially available sizing kits and invest some time in a trial at the clinic, making sure the pessary is small enough not to cause discomfort, while large enough not to be easily expelled. When initiating pessary treatment, the patient’s openness to self-management should be explored. In some cases, women prefer the assistance of a healthcare provided to periodically remove the pessary for washing (typically every 2–4 months). The availability of easily accessible professional assistance, such as a pelvic floor physiotherapy or a continence nurse, can make all the difference between successful self-management and discouragement. Common side effects of pessaries are vaginal discharge or odor. Vaginal bleeding may occur following insertion and removal because of atrophy and trauma, or from vaginal erosion or granulation tissue. Whenever the source of bleeding is not clearly identified, endometrial or cervix pathology should be considered. According to expert opinion and clinical guidelines [1,2], vaginal erosion can be managed by leaving the pessary out for 4–6 weeks and using vaginal estrogen. Once the erosion is healed, a pessary can be reinserted. More serious complications, that are rare and often associated with poor compliance, include pessary incarceration and vesicovaginal or rectovaginal fistula.

Use of estrogen cream during pessary treatment is often recommended to decrease discomfort and typical pessary complications. However, a recent large RCT [24] showed no difference in pessary continuation between post-menopausal women using vaginal estrogen cream and those using placebo (87.0% vs. 86.7%), despite a lower incidence of excessive vaginal discharge, vaginal erosion or ulcer, and vaginal bleeding, confirming previous evidence on the advantages of local estrogen [25,26]. Even in cases when pessary treatment is not acceptable as a long-term solution, a pessary trial for a few weeks may have a significant advantage, as it can help patient and provider understand whether POP reduction is effective in improving symptoms, and establish realistic expectations on the effect of surgery.

In summary, pessary treatment is a reasonable and durable option for improvement in QoL in women with symptomatic POP, with rare reports of severe adverse complications. The ideal candidate for pessary should have a good self-care index, however studies determining causative factors of the more serious adverse events are currently scarce. With appropriate counselling and professional support, good compliance and fair treatment persistence can be achieved.

### 3.3. Individually Tailored Surgical Treatment—Suggested Algorithm for a Patient-Centered Decision

Surgery constitutes the gold-standard treatment for POP. There are no absolute indications for POP surgery; however, many women, especially after completion of childbearing, seek a definitive surgical solution, and POP surgery is associated with a high rate of subjective improvement at 24 months. In the PEOPLE RCT [15], 81.5% of women in the surgery group reported subjective improvement versus 76.3% in the pessary group, and more than half of women initially assigned to pessary crossed over to surgery, often due to pessary expulsion or dissatisfaction. The aim of POP surgery is to restore normal pelvic anatomy, alleviate bothersome symptoms, improve the concomitant pelvic floor dysfunctions, and increase QoL, while minimizing recurrence and surgical morbidity. The only condition which constitutes a medical indication for POP surgery is renal failure due to hydronephrosis: the American College of Radiology [27] recognizes the association of hydronephrosis with POP which, if left untreated, can lead to permanent renal failure, and emphasizes that prompt intervention is necessary in such cases. A 2023 retrospective study [28] of 528 women with POP and hydronephrosis found that surgical intervention led to resolution of hydronephrosis in over 80% of patients, underscoring the efficacy of surgery in reversing the obstructive process.

### 3.4. Evidence-Based Considerations for Achieving Optimal Surgery Outcome

AUGS states that the choice of surgical approach should be individualized based on the location and severity of prolapse, patient comorbidities, sexual activity, and patient preference, with the goal of achieving durable anatomic support and symptom relief [3]. Reconstructive procedures aim to restore vaginal function and capacity, while obliterative procedures are considered for women who no longer desire vaginal intercourse. There are several considerations to be taken into account during surgery counselling and planning:

#### 3.4.1. Should “Perfect Anatomical Outcome” Be an Aim of Surgery?

The focus on POP surgery outcomes began to shift from primarily anatomical outcomes to subjective, patient-reported outcomes (PROs) in the late 2000s, with a clear emphasis emerging in the literature around 2009. This transition is exemplified by a pivotal study published in 2009 [29], showing that the absence of bothersome vaginal bulge symptoms was more strongly associated with patient satisfaction and perceived surgical success than strict anatomical criteria. The study found that definitions of success based on subjective cure correlated best with improvements in QoL and PRO-based success, whereas anatomical definitions alone did not capture patient experience as effectively. Subsequent research and consensus statements have reinforced this shift: the International Urogynecological Consultation (IUC) in 2023 [30] recommended that, for both research and clinical practice, surgical success should be defined primarily by the absence of bothersome patient bulge symptoms, rather than anatomical measures, and that PROs should be prioritized in both study design and clinical follow-up. Large clinical trials [15,31,32] published in the 2010s and 2020s have consistently used validated PROs as primary or co-primary endpoints, further solidifying this paradigm shift. In summary, the shift from anatomical to subjective outcomes in POP surgery consolidates the understanding that the primary surgery goal must be aligned with the patient’s functional goals and expectations, while minimizing complications and the risk of recurrence, rather than focusing on perfect anatomical outcomes.

#### 3.4.2. The Need for Appropriate Apical Support

Since the early 2000s, it has become increasingly evident that adequate apical support is the cornerstone of successful POP surgery, directly impacting anatomic restoration, symptom relief, quality of life, and recurrence rates [33]. PROs, including symptom relief and QoL, have been shown to improve after apical suspension procedures, regardless of the specific technique, with sustained benefit over time [32,34]. This paradigm shift was driven by accumulating evidence that failure to address apical support leads to recurrent prolapse, particularly in the anterior compartment, and that surgical success rates are improved with apical suspension. A pivotal study is the 2013 national cohort study by Eilber et al. [35], which demonstrated that women undergoing prolapse repair without concomitant apical suspension had significantly higher 10-year reoperation rates (20.2%) compared to those with apical support (11.6%). Subsequent RCTs and systematic reviews have reinforced this concept. The OPTIMAL trial and its 5-year follow-up confirmed that both high uterosacral ligament suspension (HUSLS) and sacrospinous ligament fixation (SSLF), procedures restoring apical support, are effective, and surgical success deteriorates over time if apical support is not addressed [32,36]. Imaging and mechanistic studies have further shown that apical descent is the primary mechanism of POP recurrence [37]. In its 2019 guideline [3], AUGS explicitly states that vaginal apex suspension should be performed at the time of hysterectomy for uterine prolapse to reduce the risk of recurrent POP, reflecting the current consensus that apical support is critical for durable outcomes.

#### 3.4.3. Should a Hysterectomy Be Part of the Surgical Plan?

According to several surveys, a substantial proportion of women prefer uterine preservation when outcomes are expected to be equivalent. In a multicenter cross-sectional study [38], 36% of women with prolapse symptoms preferred uterine preservation, 20% preferred hysterectomy, and 44% had no strong preference. However, preference for uterine preservation increased to 46% if it was described as more effective, and 21% still preferred uterine preservation even if hysterectomy was described as superior. Predictors of preference for uterine preservation included higher education, the belief that the uterus is important for self-identity and geographic region. In a further study cohort [39], 43% preferred uterine preservation and 27% preferred hysterectomy, with the remainder undecided, assuming equal outcomes. Factors influencing decision-making were expected success and risk of complications, while concerns about future cancer risk led 18% to prefer hysterectomy. Increasing evidence [40,41,42] have indicated that POP surgery with uterine preservation vs. hysterectomy yield similar outcomes, granted adequate apical support is provided: excellent long-term anatomical outcome, shorter operative time and less complications have been shown following anterior colporrhaphy and SSLF hysteropexy [40] or the Manchester-Fothergill procedure [41] vs. hysterectomy. A systematic review [42] found lower rates of reoperation for POP with hysterectomy, along with shorter operative time and decreased blood loss with uterine preservation. A recent study [43] confirm that about half of patients value the option to keep their uterus, and patient-centered, autonomy-focused approaches are increasingly advocated, emphasizing the importance of shared decision-making for women with POP.

## 4. Specific Surgical Approaches and Optimal Patient Selection

In light of the above considerations, it has become evident that there is no “one size fits all” POP surgery procedure. Regrettably, innovation in the field of pelvic floor reconstructive surgery has a tradition of discarding older surgical techniques as obsolete whenever introducing new approaches. Studies [44,45,46] conducted twenty years ago revealed that NTR surgery had 10–30% failure rates with re-operation rates as high as 50%. This led to initiatives aimed at enhancing results, by vaginal graft reinforcement or abdominal techniques. In the early 2000s, synthetic vaginal meshes for repairing POP became widely used [47,48,49,50], leading to the swift use of patented surgical kits that frequently lacked adequate training and thorough testing. Data quickly disclosed complications associated with mesh surgery, showing re-intervention rates as high as 19.7% [51], along with issues such as mesh shrinkage, erosions, dyspareunia, and uncommon but severe occurrences like structural injuries and chronic pain [52]. After issuing warnings in 2009 and 2011, the FDA mandated the halt of sales of transvaginal mesh products in 2019 [53]. The UK’s NICE introduced tougher regulations [54], following concerns raised by patient advocates about lasting harm, declaring a “high vigilance restriction period” for synthetic vaginal implants in 2018. This effectively prohibited the use of mesh, greatly limiting the treatment choices available to UK surgeons, irrespective of their clinical expertise. Although some European countries still allow the use of mesh, actions taken by the FDA and NICE have diminished its global availability and heightened concerns about potential legal consequences. While this scrutiny has successfully protected patients and surgeons from the misuse of vaginal meshes in POP surgery and its associated harms, it has effectively ‘thrown the baby out with the bathwater.’ In many regions, regulatory restrictions now prevent this approach from being offered altogether—even in cases where it remains the best available treatment option.

As the use of vaginal mesh surgery decreased, there was a significant rise in interest in abdominal sacrocolpopexy (SCP), which is now regarded as a top surgical option due to its impressive durability. Charles Nager cautioned that SCP has evolved into “a new hammer” [55]‚ a standardized solution for all types of POP, associated with more frequent, severe, and potentially life-threatening complications compared to most NTR methods. In 2023, the European Urogynecologic Association (EUGA) has issued a position statement [56] aiming to support the use of NTR as the first-line approach for surgical treatment of POP. The position statement overviewed recent evidence in safety and efficacy of NTR, its positive impact on QoL, its cost-effectiveness and sustainability. Along with its support for NTR as the first-line surgical approach for the typical woman with primary POP, the consensus highlighted the need to preserve and maintain additional surgical options for selected populations and clinical scenarios. The next paragraphs will overview the most updated evidence on the different surgical approaches available for POP, with an emphasis on patient-selection criteria for each approach.

### 4.1. Obliterative Surgery

Obliterative procedures for POP, such as colpocleisis, are highly effective, with long-term anatomic and subjective success rates exceeding 95% and low rates of regret or complications in selected patients who do not desire future vaginal intercourse. These procedures are particularly advantageous in older women with advanced prolapse and significant comorbidities, as they are associated with shorter operative times, lower perioperative morbidity, and reduced anesthetic requirements compared to reconstructive procedures [57,58]. When compared to reconstructive procedures, obliterative surgery demonstrates similar or superior efficacy in terms of prolapse resolution and QoL improvement, with some studies showing no significant difference in perioperative complication rates except for a lower risk of urinary tract infection in the obliterative group [59]. It is recognized that reconstructive procedures, while preserving vaginal function, have higher rates of recurrence and reoperation, especially with NTR, and are associated with longer hospital stays and increased perioperative risk in frail or elderly populations [59,60,61]. When considering obliterative procedures, careful patient selection is critical: this approach is reserved for women who do not desire vaginal intercourse, while reconstructive options should be the only choice for women seeking vaginal preservation [57,62]. Shared decision-making, considering patient goals and comorbidities, is essential [59,62]: some women may feel deeply disturbed by the thought of such an alteration of genital anatomy, while others may prefer it by far, given that it provides the lowest possible risk of recurrence [63]. A recent review of the literature [64] has highlighted improved body image following colpocleisis, with some women even resuming sexual activity. A crucial issue to be considered is the need to exclude risk factors for gynecologic pathologies which may require vaginal access to the cervix or uterine cavity in the future. The presence of cervical or uterine abnormalities, or risk factors for gynecological cancer, should be a relative contraindication to obliterative procedures with uterine preservation [65]. Vaginal hysterectomy can be carried out in combination with colpocleisis, however the safety advantage of colpocleisis is lost with such a combination [66]. In summary, obliterative procedures are at least as effective as reconstructive procedures for prolapse resolution and QoL, and should be considered a legitimate choice to avoid perioperative risk in the appropriate population.

### 4.2. Vaginal Native Tissue Reconstructive Surgery

POP surgery by NTR continues to be the first-line surgical choice for primary POP, as it offers a good combination of effectiveness, safety, and minimizes the risk of complications associated with mesh use. A Cochrane review from 2024 [67], revealed that while transvaginal permanent mesh results in somewhat lower rates of prolapse awareness and repeat surgeries for POP compared to NTR, it also poses greater risks for total repeat surgeries (including those related to mesh exposure), bladder injuries, and the onset of new stress urinary incontinence. The meta-analysis concluded that the balance of risks and benefits does not justify the routine use of mesh in primary surgeries, but rather synthetic mesh should be utilized in carefully chosen instances and with stringent supervision [67]. RCTs and prospective cohort studies [68,69,70,71] with follow-up of 3–6 years have demonstrated that NTR yields comparable subjective and functional results to mesh or biological graft augmentation. There are no significant differences noted in patient-reported prolapse symptoms, rates of reoperation, or sexual function. Mesh and grafts do not enhance long-term cure rates and come with specific risks, such as mesh exposure and the potential necessity for reoperation related to the mesh. Current developments in surgical practices indicate a growing preference for native tissue repairs and uterine-preserving techniques, resulting in fewer perioperative complications and shorter hospital admissions [72]. Uterine-preserving NTR is recognized as a safe and effective alternative to hysterectomy, aligning with patient choices and in some studies showing a reduced risk of anatomical recurrence and complications at 1-year follow-up [43]. In conclusion, NTR is widely regarded as the preferred treatment for primary POP, backed by strong evidence demonstrating its safety, effectiveness, and positive patient outcomes.

### 4.3. Mesh-Augmented Vaginal Repair

The rationale for using transvaginal mesh in POP repair for selected patients is its durability. Unlike NTR, permanent transvaginal mesh decreases the chances of anatomical recurrence and reduces postoperative POP awareness, along with lower rates of reoperation for recurrent POP [67]. However, this advantage comes with the potential for specific mesh-associated risks and an overall increase in reoperations for mesh-related complications [73]. According to the latest Cochrane meta-analysis [67], permanent mesh resulted in reduced POP awareness (RR = 0.83) and a lower rate of reoperation for POP (RR = 0.71) compared to NTR. Nevertheless, it was associated with a higher overall rate of repeat surgeries due to mesh exposure, as well as increased risks of bladder injury and new cases of SUI. Mesh exposure was observed in approximately 12% of cases, with about 6% needing surgical intervention. Recent registry data [73] indicate that transvaginal mesh results in fewer repeat surgeries for recurrence compared to NTR, although it is associated with a higher incidence of serious complications, yet the complication rates is similar to that seen with laparoscopic SCP.

Appropriate candidates and clinical scenarios for vaginal mesh-augmented repair include:

○ Recurrent prolapse, particularly in the anterior or apical regions, following failed NTR, where further NTR durability is a concern. The American College of Obstetricians and Gynecologists advises that vaginal mesh for POP “should only be used in high-risk patients,” such as those experiencing recurrent POP, especially in the presence of morbidity that make more invasive abdominal or endoscopic procedures unsuitable [74].
○ Patients with significant anterior or apical defects for whom an abdominal approach (such as sacrocolpopexy) is not advisable or possible may be treated by skilled surgeons utilizing lightweight, lower-load apical-only devices, while also being informed about the risks of mesh exposure and the potential for new de-novo SUI [34].
○ Selected patients opting for uterus-sparing hysteropexy where mesh-augmented apical support may improve anatomic durability with low short-term exposure rates, in jurisdictions where permitted and by high-volume teams [75]. Women who may significantly benefit from vaginal mesh-augmented repair in this scenario include 2 groups:
  ○ Women with advanced anterior wall stage 3 or stage 4 POP [76,77,78].
  ○ Women with sonographic evidence of levator ani muscle avulsion [79].

In the above clinical scenarios, following appropriate counselling on risks and benefits of each approach, and in the hands of experienced surgical teams, mesh-augmented repair has been shown to provide lower rates of subjective and objective POP recurrence [67,80,81].

### 4.4. Minimally Invasive Abdominal Approach

Sacrocolpopexy (SCP) is regarded as the gold-standard procedure for apical POP repair. Compared to vaginal NTR suspensions, SCP offers better anatomical durability and symptom relief, leads to fewer repeated POP surgeries, less postoperative stress incontinence, and has similar or lower dyspareunia rates. Moderate-certainty randomized data compiled by the latest Cochrane review on POP surgery indicate lower awareness of prolapse (RR = 0.43), reduced anatomical recurrence (RR = 0.53), and fewer reoperations (RR = 0.43) following SCP compared to vaginal procedures. Laparoscopic SCP results in shorter hospital stays than open SCP and is quicker than robotic SCP in RCTs [33]. A recent RCT enrolling patients undergoing apical repair for vaginal vault prolapse demonstrated that SCP was associated with lower failure rates compared to NTR [82]. These findings are in contrast to the evidence presented by the authors of the SALTO-2 study, who found no significant difference in outcomes when comparing native tissue repair of the SCP (specifically, vaginal sacrospinous fixation). Therefore, it is not yet possible to provide conclusive evidence on what the true gold standard for surgical treatment of apical prolapse is. Nevertheless, SCP should not be the primary choice for the typical POP patient due to associated trade-offs such as mesh-related risks (with late erosions occurring in 2–3% or more, especially when combined with total hysterectomy), longer surgery duration, increased resource consumption (particularly with robotic-assisted SCP), and similar patient-reported outcomes when compared to certain vaginal NTR [33,83,84,85].

In the following clinical scenarios, SCP’s advantages outweigh risks and is fully indicated:

- Post-hysterectomy apical prolapse desiring maximal anatomic durability/length preservation and sexual function optimization [33,83,86].
- Multicompartment prolapse where a single apical suspension with paravaginal support can address global defects; nerve sparing SCP may reduce need for posterior repair and improves bowel symptoms [87].
- Recurrent apical prolapse after failed vaginal native-tissue repair [33,85].
- Younger/sexually active patients prioritizing vaginal length and axis; SCP maintains total vaginal length better than some vaginal hysterectomy-based approaches [33].

Pectopexy, a procedure using synthetic mesh anchored to the bilateral pectineal ligaments, was first introduced in 2011 [88]. This procedure is thought to have a lower risk of complications since it focuses solely on the front portion of the pelvis, thereby reducing the likelihood of harming nearby organs. Despite current level of evidence of low to moderate quality [89], pectopexy may be a reasonable alternative in selected patients with concerns about sacral dissection or bowel dysfunction, with similar anatomic outcomes and less postoperative bowel dysfunction in pooled analyses [90,91].

### 4.5. Evidence on Safety and Efficacy of Different Surgical Approaches According to Vaginal Segment/Level of Support

#### 4.5.1. Level 1—Apical Prolapse Repair

A Cochrane review [92] on apical prolapse surgery found SCP superior to NTR regarding prolapse awareness, repeat surgery, examination findings, and dyspareunia. Evidence showed no transvaginal mesh superiority over NTR, establishing SCP as the gold-standard for apical suspension. However, a recent meta-analysis [93], comparing SCP to SSLF concluded that while SCP offers better durability and SF, SSLF avoids mesh erosion and has shorter operative time, fewer gastrointestinal complications, less hemorrhage, and fewer wound infections. Another meta-analysis [94] comparing vaginal and abdominal apical repair approaches found similar subjective outcomes and complications at 1–5 years, confirming vaginal NTR techniques provide satisfactory apical suspension. A recent RCT [95] comparing SSLF to the Manchester procedure has shown better composite outcome for the latter (77% vs. 87.3%, composite success, respectively. *p* = 0.007), a result mostly related to higher rates of recurrent cystocele with SSLF.

#### 4.5.2. Level 2—Anterior Vaginal Prolapse Repair

The primary motivation for vaginal mesh surgery was the unsatisfactory outcome of native tissue anterior colporrhaphy. However, vaginal meshes proved less safe and effective than initially thought when used in unselected cohorts. The most recent Cochrane review [67] found that transvaginal permanent mesh has lower rates of prolapse awareness, repeat prolapse surgery, and prolapse on examination than NTR, but higher rates of total repeat surgery (for prolapse, stress urinary incontinence, or mesh exposure), bladder injury, and de novo stress urinary incontinence. Therefore, careful risk-benefit discussion should restrict vaginal mesh use to women at high recurrence risk: those with advanced stage 3/4 POP, levator ani avulsion, or symptomatic recurrent cystocele. For severe combined anterior and apical POP, recent evidence [96] has indicated that laparoscopic lateral suspension with mesh yields high objective (88.3%) and subjective (93.3%) success, along with a high safety profile. Chmielewski et al. [97] reanalyzed Weber et al.’s [48] study comparing three AC methods: standard colporrhaphy, standard colporrhaphy plus mesh, and ultralateral AC. The results of this repeated analysis supports the efficacy of NTR anterior colporrhaphy for primary cystocele: while original anatomy-based success rates were 30%, 42%, and 46% respectively, assessment using clinically relevant criteria yielded 88% success with no group differences and only one repeat surgery for recurrence.

#### 4.5.3. Level 2—Posterior Vaginal Prolapse Repair

A Cochrane meta-analysis [98] on posterior compartment prolapse recommended transvaginal NTR as the best approach for rectocele repair, concluding that evidence does not support using mesh for posterior vaginal repair. Regarding posterior compartment outcomes following SCP, large studies show persistent/recurrent rectocele is common [99,100,101], though whether adding posterior colporrhaphy (PC) improves outcomes remains unclear. Some studies [101,102] indicate concomitant PC provides more durable anatomical support [102,103] and reduces composite patient-centered failure without causing dyspareunia or defecatory dysfunction [101]. Others [87,100,104] found similar posterior vaginal support improvement following SCP with or without PC. In summary, women with symptomatic rectocele benefit most from plication of posterior fibro-vascular connective tissue, with outcomes further improved by addressing apical defects [35].

#### 4.5.4. Level 3—Repair of a Wide Genital Hiatus (GH)

Vaginal laxity is a poorly defined condition often coexisting with POP, possibly representing early-stage prolapse. While apical suspension can reduce GH width [105], some SCP studies advocate concomitant perineorrhaphy for level 3 defects, as this may decrease early composite anatomic failure [99]. However, whether perineorrhaphy consistently improves sexual satisfaction remains unclear, and mild pelvic floor relaxation may benefit sexual function [29], especially in post-menopausal women. In women complaining of “widened vagina” sensation, evidence suggests perineorrhaphy, a level 3 NTR technique, provides high patient satisfaction with few complications [106,107]. Genital hiatus surgery should be carefully considered during POP surgery, with surgeons discussing potential perineorrhaphy risks and benefits with patients [108].

## 5. Conclusions

As the female population ages and enjoys improved overall health, healthcare providers are encountering an increasing need to address QoL issues like POP. The key to effective treatment is adopting a patient-focused, personalized strategy that takes into account the unique goals, preferences, and expectations of each woman. In recent decades, the definitions of treatment success have changed considerably, moving from a focus solely on anatomical results to placing more importance on PROs and functional enhancements. Going forward, it is essential to pair ongoing advancements in surgical methods with careful patient selection to avoid overtreatment and decrease the risk of complications. Clinicians face the challenge of selecting the most suitable intervention for each patient by taking into account not just the severity of anatomical defects, but also the burden of symptoms, functional effects, and the patient’s personal treatment objectives. This tailored approach guarantees that women receive treatment that genuinely enhances their QoL while minimizing unnecessary hazards.

## Data Availability

Not applicable.
